# Does the establishment of Pilot Free Trade Zones promote international expansion of enterprises? Quasi-natural experimental evidence from China

**DOI:** 10.1371/journal.pone.0308477

**Published:** 2024-08-15

**Authors:** Wenqi Jing, Yi Zheng, Xiuqing Shen

**Affiliations:** School of Business, Inner Mongolia University of Finance and Economics, Hohhot, Inner Mongolia, China; Qingdao University, CHINA

## Abstract

Pilot Free Trade Zones (PFTZs) are a crucial new platform for China to build a more open economic system. Existing literature primarily focuses on the ‘Bring In’ effect of PFTZs, often overlooking the importance of ‘Going Out’ aspects. To bridge this gap, this paper uses data from China’s Shanghai and Shenzhen A-share listed enterprises from 2007 to 2021 and constructs a time-varying difference-in-differences (DID) model to test the impact of establishment of PFTZs on international expansion of enterprises. The study finds that establishment of PFTZs can significantly promote international expansion of enterprises, with a more pronounced effect on the scope of international expansion than on its depth. Mechanism analysis reveals that PFTZs can facilitate international expansion of enterprises by driving digital transformation, enhancing total factor productivity and management efficiency, and alleviating financing constraints. Notably, senior managers with overseas work experience play a crucial role in enhancing this relationship. Further, PFTZs not only have a linkage effect with the Belt and Road Initiative but also a radiation effect on neighboring cities. This study provides an analytical perspective and empirical evidence for evaluating policy effects of PFTZs and offers valuable insights that will enable PFTZ policies to be refined and facilitate successful implementation of the ‘Going Out’ strategy.

## 1. Introduction

International expansion is a crucial strategic decision for enterprise development [1]. In the 21st century, the dominant position of traditional multinational enterprises from developed economies in the international market is undergoing profound changes with the rise of emerging economies [2–4]. Taking the largest emerging economy, China, as an example, its enterprises are transitioning from passive involvement, in which they were primarily brought into globalization by multinational corporations through capital investment, technology transfer, and order outsourcing [5], to more active integration into international markets and participation in global competition. However, with the global COVID-19 pandemic, along with increasing international political volatility and frequent trade frictions, has significantly heightened the external risks and uncertainties faced by enterprises in their international expansion efforts. Moreover, enterprises themselves encounter various challenges in internationalization, including technology blockades [6], financing difficulties [7, 8], and a shortage of experienced international talents [9], leading to weak international competitiveness and insufficient motivation to further expand into overseas markets. Given these circumstances, it is imperative to explore how to overcome these obstacles to facilitate the international expansion of enterprises, which is not only conducive to the growth of enterprises but also holds great significance in enhancing China’s domestic-international economic dual-circulation development pattern.

Current academic research on international expansion primarily focuses on internal enterprises characteristics, such as executive background [10], technological innovation [11]. However, there is a lack of discussion on the influence of external policy shocks. Institutional economics emphasizes the significance of an effective institutional system for economic development [12]. In this context, Pilot Free Trade Zones (PFTZs) represent a significant institutional innovation in China, aiming to create a new platform for comprehensive opening up and promote high-quality development. From September 2013 to November 2023, China established 22 PFTZs in seven batches across the nation (see, S1 Table), with the objective of fostering a more comprehensive open economic environment. Existing research on PFTZs has primarily focused on the ‘Bring In’ perspective, including attract foreign investment, promote import trade, and drive regional economic growth [7, 13, 14]. However, there is a research gap in the literature regarding the impact of PFTZs on international expansion of enterprises from the ‘Going Out’ perspective, which requires more attention.

As national-level ‘experimental fields’, PFTZs not only provide an efficient platform for domestic and foreign enterprises to carry out international exchanges, but they also have the capacity to attract global resources, which cluster within PFTZs. This clustering enables domestic enterprises to better utilize and integrate these resources, thereby deepening their participation in global economic and trade cooperation and creating favorable conditions for enhancing their competitiveness in the international market. Thus, PFTZs can provide a strong institutional guarantee for enterprises to develop new comparative and competitive advantages in the fiercely competitive international market.

Building on this discussion, we employ China’s A-share listed enterprise data and construct a difference-in-differences (DID) model by treating the establishment of PFTZs as a quasi-natural experiment, to assess the impact of PFTZs on international expansion of enterprises, and explore the potential mechanisms of the impact from the perspective of ‘Going Out’. This study not only enhances understanding of the microeconomic effects of PFTZs form different perspectives, but also provides new insights for optimizing resource allocation in enterprise factor markets and promoting China’s high-quality economic development.

This study supplements the existing literature with several novel insights. First, this study fills a research gap by being the first to scrutinize the policy effect of PFTZs on international expansion of Chinese enterprises. Previous studies have paid attention to impact of the establishment of PFTZs from the ‘Bring In’ perspective [13, 14], but have neglected to consider its impact on ‘Going Out’ perspective. Our study expands the quantitative analysis of PFTZs’ microeconomic effects from the ‘Going Out’ perspective, which opens up a way for future research to evaluate policy effect of the PFTZs. Second, we treat PFTZ as a quasi-natural experiment and group them at the city level, with pilot cities as the treatment group and non-pilot cities as the control group, which allows us to identify the causality between PFTZs and international expansion of enterprises using the DID method. By doing so, our study provides a more detailed understanding of the extent of PFTZ implementation compared to previous research [15], which offers a solid factual basis for evaluating the economic consequence of PFTZs. Third, our study contributes to existing research by providing empirical evidence that supports the impact of PFTZs on international expansion of enterprises in terms of both depth and scope dimensions. Moreover, we shed light on the key channel mechanisms through which PFTZs facilitate international expansion, including the technology effect, competition effect, and resource allocation effect. Finally, we explore the role of PFTZs in serving national strategies and fostering coordinated regional development. In recent years, a number of studies have explored the linkage effect between the establishment of PFTZs and the Belt and Road Initiative (BRI) in various contexts [16]. Our analysis adds to this literature by revealing their linkage effect on international expansion of enterprises, as well as its radiation effect on neighboring non-pilot cities.

The reminder of the paper is organized as follows. Section 2 reviews the literature. Section 3 presents the policy background, mechanism analysis, and theoretical hypotheses. Section 4 constructs the econometric model and describes the variable data and sources. Section 5 tests the theoretical hypotheses and expansion analysis. Conclusions and policy implications in Section 6.

## 2. Literature review

Establishment of PFTZs is an important practice for institutional innovation in the field of deepening reform and opening up. It serves as a new platform for China’s high-level opening up in the new era, and holds great significance for China’s economy to achieve high-quality development. In recent years, as development of PFTZs has progressed, the economic effects of PFTZs have received widespread attention in the academic community. From a macro perspective, research on PFTZs has mostly focused on regional economic growth [13, 17], the import-export trade [14], foreign direct investment [7], and regional innovation capacity [18]. For instance, [19] used the SPFTZ as a case study, and discovered that the establishment of SPFTZ has alleviated capital governance and accelerated financial liberalization. [20] conducted an assessment of the economic effects of China’s coastal PFTZs using the grey relational analysis method, showing that the PFTZs had a significantly higher economic growth promotion effect on the Yangtze River Delta and the Bohai Rim Economic Zone than other regions. [21] showed that the establishment of PFTZs can significantly reduce trade costs, thereby enhancing trade efficiency. From a micro perspective, research is relatively limited and primarily focuses on firm performance [22], technological innovation [23], productivity [24], and optimization of human capital structure [25, 26]. [22] conducted a study on 16 listed port companies and found that establishment of PFTZs can significantly improve the operational performance of these companies. Furthermore, this promotion effect has a sustained impact.

In terms of the international expansion of enterprises, research shows that scholars are primarily interested in factors affecting the internationalization of enterprises. Existing research mainly focuses on studying the influencing factors of international expansion of enterprises. From the perspective of the international environment, existing studies have confirmed that significant cultural differences [27, 28] and high external environmental uncertainty [29] inhibit international development of enterprises. Additionally, the legal environment of the host country [30] is also an important factor for corporate decision-makers to consider when adopting internationalization strategies. From the perspective of technological advancements, digital transformation can help reduce transaction costs such as transportation and communication in transnational business activities, thereby improving cooperation efficiency [31–33]. Simultaneously, it enhances the information processing ability of enterprises and enables them to accumulate relevant knowledge and experience to gain a deeper understanding of the international market [34]. Additionally, green technology innovation can also can significantly increase the export of enterprises [35]. From the perspective of corporate governance, ownership nature [36, 37], executive characteristics [38], and foreign ownership [39] can also have different effects on the international expansion of enterprises. For example, [37] found that the internationalization strategy of state-owned enterprises is driven by national strategic objectives. These enterprises tend to choose more challenging countries and use M&A schemes more intensively, especially under unfavorable market conditions. Additionally, executives with international experience in the company are more inclined to adopt internationalization strategies when making decisions [38].

Upon reviewing the literature, it becomes evident that scholars have conducted extensive research on PFTZs, contributing to understanding the role of the construction of PFTZs in further expanding China’s opening-up and deepening economic system reforms. Nevertheless, there are still some deficiencies that need to be addressed. First, existing literature has not systematically differentiated between the depth and scope of enterprises’ international expansion, which hinders a detailed understanding of the underlying mechanisms by which PFTZs influence enterprises’ international expansion. Second, previous studies have often chosen provincial-level samples, treating the entire province as a PFTZ. While this approach allows for examination of the regional characteristics and overall effects of PFTZs, it does not consider the fact that only a few specific cities within each province are designated as PFTZs. Consequently, conducting research at the provincial level may not reveal the micro mechanisms behind these effects, potentially leading to estimation biases when assessing the policy effects of establishment of PFTZs. The primary objective of both PFTZs and the BRI is to promote trade and investment liberalization. In principle, the overlapping impact of these two policies could potentially deliver greater advantages for international expansion of China’s enterprises. However, existing literature has not thoroughly explored the policy interaction effect of PFTZs and the BRI. This study aims to address the aforementioned research gap.

## 3. Policy background, mechanism analysis and theoretical hypotheses

### 3.1. Policy background

Establishment of PFTZs represents a major strategic initiative for China in establishing a new platform for comprehensive opening up, representing an apex in institutional innovation, and leading to high-quality development as a new carrier. Since China’s first PFTZ, the Shanghai Pilot Free Trade Zone (SPFTZ), was established in 2013, China has approved 22 PFTZs in seven batches; this initially formed an open situation of regional coordination and land-sea coordination, providing a solid foundation and strong and lasting impetus for promoting high-quality development of China’s economy. Over the past decade, with institutional innovation at its core, establishment of PFTZs has undertaken the role of ‘being prior to carry and try’, launching a series of pioneering practices that have been involved in accelerating transformation of government functions [15], promoting facilitation of investment and trade [14], optimizing the management system for foreign investment and reforming the financial service system [19]. Numerous reformative and opening-up institutional innovations have been replicated and promoted nationwide, effectively demonstrating the role of PFTZs as an ‘experimental fields’ for reform and opening up.

### 3.2. Mechanism analysis and theoretical hypotheses

#### 3.2.1. Direct impacts of the establishment of PFTZs on international expansion of enterprises

In recent years, the international market environment has become increasingly volatile and uncertain. This has amplified the risks faced by enterprises in their international expansion efforts, and affected their willingness to adopt international expansion strategies [40]. In this background, the establishment of PFTZs, which are an institutional innovation, provides a strong institutional basis for international expansion of enterprises. According to the theory of institutional economics, the institutional environment can have a significant impact on business activities and performance of enterprises [12, 41]. Establishment of PFTZs offers a favorable institutional environment for promotion of trade liberalization and investment facilitation. This is achieved through a variety of measures that include reducing trade barriers, expediting customs clearance, reforming exchange rate mechanisms and adopting negative list management systems. PFTZs encourage the smooth and unrestricted flow of domestic and international production factors, efficient resource allocation and deep market integration, all of which are conducive to accelerating the construction of a modern market system aligned with international rules. As a result, establishment of PFTZs not only attracts substantial foreign investment but also stimulates growth in China’s export-oriented industries. Moreover, PFTZs support the ‘Going Out’ strategy of Chinese enterprises. They serve as platforms for China’s enterprises to expand their international influence, enter global markets and forge strategic partnerships with foreign enterprises. Therefore, this paper puts forward Hypothesis 1.

H1: Establishment of PFTZs can promote international expansion of enterprises.

In addition, based on the research logic of [24] we discuss the mechanism driving the effect of establishing PFTZs on international expansion of enterprises from three perspectives: the technology effect, the competition effect and the resource allocation effect.

#### 3.2.2. Mechanism for the technology effect

According to endogenous economic growth theory, technological differences are the fundamental causes of development gaps [12, 41]. Currently, a new generation of information technology, i.e. digital technology, is driving substantial transformation in global economy. Integrating digital technology has injected fresh impetus into enterprise development, leading to disruption of organizational structures, business performance, strategic choices and business models [42–44]. On the one hand, PFTZs provide enterprises with a broader development space by exploiting superior institutional advantages and high-level production factors. This attracts domestic and international high-tech and innovative companies to particular zones, promoting geographical clustering of digital industry within PFTZs. As a result, it greatly increases the degree of collaboration between enterprises within zones [31], fostering closer exchange of digital technology [8]. On the other hand, applying digital technology can significantly decrease transaction costs related to information searching, cross-border communication and logistics transportation [32]. It can also enhance the information perception ability of enterprises within complex international markets. These help to overcome the problem of information asymmetry in the international market [34], providing a technical foundation for enterprises’ international expansion. As a result, this paper proposes Hypothesis 2.

H2: Establishment of PFTZs can promote international expansion of enterprises by facilitating digital transformation.

#### 3.2.3. Mechanism for the competition effect

PFTZs have effectively weakened entry barriers for foreign-funded enterprises and export restrictions for local enterprises through implementation of pre-establishment national treatment and negative list management modes [45]. Furthermore, transition from an examination and approval system to a registration and recording system has also substantially enhanced operational efficiency for businesses. Considerable policy advantages associated with PFTZs can attract large numbers of high-quality domestic and international enterprises to invest and establish a presence; this results in the share of local enterprises in local markets being squeezed, and enterprises in PFTZs facing a more intensely competitive environment [46]. Under these circumstances, enterprises must enhance productivity and optimize organizational management if they are to withstand fierce market competition. This market-based selection mechanism, favoring survival of the fittest, helps to force improvements in total factor productivity and management efficiency. As total factor productivity and management efficiency continues to improve, the competitiveness of enterprises is significantly enhanced. In pursuit of broader markets and maximized profits, enterprises may actively expand overseas [47]. In summary, the agglomeration effect generated by PFTZs promotes intensified competition amongst enterprises, compelling them to enhance total factor productivity and management efficiency to facilitate entry into the international market. Therefore, we propose Hypothesis 3.

H3: Establishment of PFTZs can promote international expansion of enterprises by improving total factor productivity and management efficiency.

#### 3.2.4. Mechanism for the resource allocation effect

In terms of financial resource allocation, constraints often pose a substantial obstacle to international expansion of enterprises. Firstly, the investment facilitation effect of PFTZs may facilitate the ease with which financial capital flows into enterprises within pilot zones, thereby alleviating financing constraints [8]. Specifically, establishment of PFTZs has promoted financial openness and innovation, relaxing stringent controls on private capital, foreign institutions and venture capital firms in the financial services sector. These entities are encouraged and supported to engage in operational activities within PFTZs, ensuring a necessary capital supply for the international expansion of enterprises in the region. Secondly, establishment of the free trade account system has effectively addressed the limitations on capital account transactions [19]. With the free trade account system, enterprises can carry out local currency payment and settlement for cross-border transactions, as well as engage in cross-border exchange and overseas business financing. Thus, the free trade account system promotes convenience in import-export trade and investment-financing; and reduces the trade settlement cost and financing cost of enterprises [7].

In terms of talent resource allocation, a lack of overseas experience has emerged as a significant factor constraining international expansion of enterprises. The upper echelons theory holds that strategic decisions made by companies are influenced by the psychological characteristics of senior managers, including their cognitive abilities and values [48]. Therefore, personal characteristics of senior managers play a pivotal role in the strategic decision-making of enterprises [49]. Consequently, introduction of senior managers with overseas work experience can bring a wealth of intangible resources to enterprises, including valuable knowledge, experience and international social networks [38]. These resources can help enterprises overcome external challenges during international expansion such as information asymmetry [50], cultural barriers [9] and institutional constraints [38]. In summary, we propose Hypothesis 4a and 4b.

H4a: Establishment of PFTZs can promote international expansion of enterprises by alleviating financing constraints.
H4b: Senior managers with overseas work experience can enhance the positive effect of establishing PFTZs on international expansion of enterprises.

**Fig 1:**
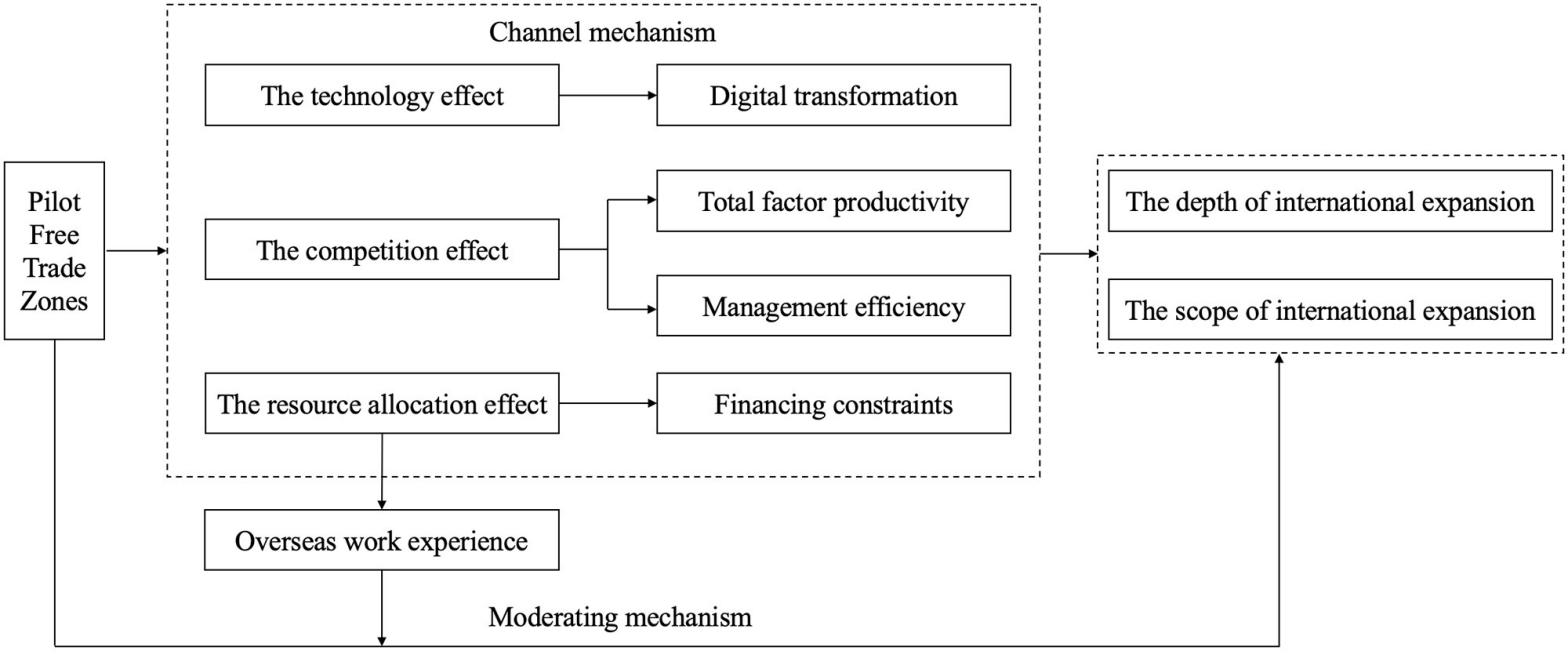
The impact mechanisms for the role of establishing PFTZs on international expansion of enterprises.

In summary, this paper presents a framework (Fig 1) that illustrates the impact mechanisms for how the establishment of PFTZs affects international expansion of enterprises.

## 4. Study design and data description

### 4.1. Regression model

DID method has been widely applied in recent years to evaluate policy effects [51]. Its principle is to regard the implementation of new policies as a quasi-natural experiment that occurs externally to the economic system, and to evaluate any changes for observed factors (e.g., policy occurrence or non-occurrence) by constructing a counterfactual framework. Theoretically, selection of PFTZs is generally not influenced by micro enterprises, which overcomes reverse causality and accurately identifies the net impact of establishment of PFTZs on the international expansion of enterprises [52]. Hence, in this study, it is appropriate to adopt the DID method to evaluate policy effects of PFTZs. Considering that establishment of PFTZs has been launched in multiple phases, we constructed the following time-varying DID model Eq (1).

$$\begin{array}{c} DEPTH_{it}/SCOPE_{it}=\beta_{0}+\beta_{1}PFTZ_{ct}+\beta_{2}X_{ict}+\gamma_{i}+\mu_{t}+\eta_{j}+\varepsilon_{it}, \end{array}$$ (1)

Where *DEPTH_it_* and *SCOPE_it_* represent the depth and scope of international expansion of enterprise *i* in year *t*, respectively. The core explanatory variable *PFTZ*_*ct*_ is defined as a dummy variable, which is assigned to 1 if PFTZ has been established by the city *c* in year *t*, and 0 otherwise. *X*_*ict*_ denotes a series of the vector of firm-level and city-level control variables. *γ*_*i*_, *μ*_*t*_, and *η*_*j*_ denote the year, firm, and industry fixed effects, respectively. *β*_*0*_, *β*_*1*_ and *β*_*2*_ are the parameters to be estimated, respectively. *ε*_*it*_ is the random error term. *β*_*1*_ is the coefficient of primary interest in this paper, as it captures the net effect of establishment of PFTZs on the international expansion of enterprises.

### 4.2. Variable selection

#### 4.2.1. Dependent variables

This study systematically divides the international expansion strategy of enterprises into two dimensions: depth and scope. The depth of international expansion of enterprises (DEPTH) reflects the overseas operational performance of that enterprise. In this paper, the proportion of overseas sales revenue to total sales revenue is used as an indicator measure of DEPTH [53]. The scope of international expansion of enterprises (SCOPE) reflects the diversity and complexity of overseas investment in that enterprise, with the most representative measures including the number of countries where overseas subsidiaries are located and the total number of overseas subsidiaries [54]. In this paper, the natural logarithm of the number of different overseas countries in which the enterprise invests, is used as an indicator measure of SCOPE.

#### 4.2.2. Independent variable

In 2013, China established its first PFTZ, known as the SPFTZ. This pioneering move aimed to explore a new institution that fosters openness and economic growth [19]. Success of the SPFTZ prompted China to further expand this model, resulting in establishment of a total of 22 PFTZs across the country to date. However, the sample interval for this paper covers the years from 2007 to 2021, while the sixth batch of PFTZs was only established in 2020; this means that the observation period for the sixth batch of PFTZs is relatively short within the sample. For this reason, our study focuses on examining the first five batches of PFTZs (see, S1 Table), thereby ensuring a more comprehensive and representative analysis.

At present, most studies have considered where the PFTZs are located as the treatment group samples at the province level, while others serve as control group samples. However, it should be noted that, except for Hainan Province, implementation of PFTZs in other provinces takes the form of specific zones within cities. To enhance the reliability of estimation results, treatment group samples are defined as the cities where the implementation zones of PFTZs are located within each province, while the remaining areas serve as control group samples. Specifically, if city *c* is approved as a PFTZ in year *t*, the variable PFTZ is assigned to 1, and 0 otherwise.

#### 4.2.3. Mechanism variables

According to mechanism analysis, we examine the impact mechanism of PFTZs on international expansion of enterprises from three aspects: technology effect, competition effect and resource allocation effect. First, the technological effect is represented by digital transformation (DT). Digital technology significantly enhances the efficiency of enterprise information processing and circulation, accumulates greater innovation potential, thus accelerate digital transformation can promote development of enterprises. Based on the research of [55, 56], we employ Python text mining capabilities to analyze and summarize the frequency of digital transformation-related terms in the annual reports of sample companies, then add 1 to the count and take the natural logarithm to measure DT. Second, the competition effect is expressed in terms of enterprise’s total factor productivity (TFP) and management efficiency (ME). Following the approach proposed by [57], we use the OP method to estimate the natural logarithmic TFP of enterprises. ME is measured by calculating the inverse of the ratio of management expenses to total income [22]. Third, we examine the resource allocation effect of enterprises from the perspectives of capital and talent. The resource allocation effect is represented by enterprises’ financing constraints (FC) and senior managers with overseas work experience (OWE). FC is calculated based on the approach introduced by [58]. OWE is measured by the ratio of senior managers with overseas work experience to the total number of senior managers, reflecting the talent resources.

#### 4.2.4. Control variables

Referring to [15, 16, 22, 59], we chose the following indicators as firm-level and city-level control variables: (1) enterprise size (Size), which is the natural logarithm of the total assets of the enterprise at the end of the year; (2) enterprise age (Age), which is the natural logarithm of the difference between the observation year and founding year; (3) return on equity (ROE), which is the ratio of net profit to total assets of the enterprise; (4) cash flow ratio (Cashflow), which is the ratio of cash holdings to total assets of the enterprise; (5) revenue growth rate (Growth), which is the annual growth rate of operating revenue of the enterprise; (6) ownership concentration (Top1), which is the proportion of shares held by the largest shareholder; (7) per capita gross domestic product (PGDP), which is the ratio of regional gross domestic product to population; (8) government intervention (GI), which is the proportion of general budget spending at the city level to GDP; (9) financial development level (FL), which is the balance of deposits and loans of financial institutions as a share of GDP at the end of the year; (10) industrial structure (IS), which is the ratio of the value-added of the tertiary sector to the value-added of the secondary sector; (11) opening degree (OD), which is the ratio of total import and export trade to GDP.

### 4.4. Data source

This study selects Chinese A-share listed enterprises in Shanghai and Shenzhen from 2007 to 2021 as the research sample. The sample and data for enterprises are obtained from the China Stock Market and Accounting Research Database (CSMAR), and the digital transformation data comes from the annual reports of the enterprise. Prefecture-level cities data comes from the China City Statistical Yearbook. To mitigate the impact of outlier samples, we clean the original data using the following steps: (1) excluding enterprises with ‘ST’ and ‘*ST’ in the stock codes; (2) excluding enterprises in the financial industry, such as banks, insurances and securities; (3) retaining enterprises without missing data for at least 2 consecutive years; (4) excluding enterprises with severe missing values for key variables; (5) winsorizing all continuous variables at the 1% level. After applying the above steps, a total of 24,159 observations from 2,742 enterprises in 225 cities are obtained for further analysis. Table 1 displays the descriptive statistics of the main variables after processing. It shows that the mean value and standard deviation of DEPTH were 0.0462 and 0.1398, respectively. These values indicate a significant variation in DEPTH among enterprises. The minimum and maximum values of DEPTH range from 0 to 1, representing the range from no reliance to complete reliance on overseas markets.

**Table 1:** Descriptive statistics of variables.

VarName	N	Mean	SD	Min	Max
DEPTH	24159	0.0462	0.1398	0.0000	1.0000
SCOPE	24159	0.4334	0.6146	0.0000	3.6376
PFTZ	24159	0.2401	0.4272	0.0000	1.0000
Size	24159	22.1728	1.2883	19.4058	26.4297
Age	24159	2.8261	0.3587	0.6931	3.6109
Roe	24159	0.0685	0.1259	-1.0721	0.4464
Cashflow	24159	0.0478	0.0699	-0.2244	0.2825
Growth	24159	0.1871	0.4241	-0.6597	4.3304
Top1	24159	0.3507	0.1496	0.0813	0.7584
PGDP	24159	11.2997	0.5951	8.5993	13.0557
GI	24159	0.1541	0.0564	0.0437	1.0268
FL	24159	1.5818	0.6598	0.0753	7.4502
OD	24159	0.6074	0.6053	0.0001	3.6397
IS	24159	0.4216	0.1104	0.1170	0.9097
DT	24159	1.2030	1.3897	0.0000	6.3063
TFP	24159	9.0949	1.1177	5.7754	13.5560
ME	24159	-0.0888	0.0719	-0.7660	-0.0068
SA	24159	-3.7573	0.2673	-5.2368	-2.1126
OWE	24159	0.0568	0.1000	0.0000	0.8889

## 5. Empirical results and analysis

### 5.1. Benchmark regressions

Table 2 reports benchmark regression results for the impact of establishment of PFTZs on international expansion of enterprises (i.e., DEPTH and SCOPE). Columns (1)—(3) focus on the policy effect of PFTZs on Depth. The results show that after gradually adding firm-level and city-level control variables in columns (2) and (3), the coefficients of PFTZ remain significantly positive. Columns (4)—(6) focus on the policy effect of PFTZs on SCOPE. All coefficients of PFTZ in Columns (1)—(6) are significantly positive at a least 5% level, which suggests a clear causal relationship between establishment of PFTZs and international expansion of enterprises. Therefore, H1 is preliminarily supported; that is, when other conditions remain unchanged, the establishment of PFTZs can significantly promote international expansion of enterprises. Furthermore, it is noteworthy that there is a substantial difference in the coefficients of PFTZ between Columns (3) and (6), indicating that the impact of establishment of PFTZs is more pronounced on the DEPTH than on its SCOPE.

**Table 2:** Benchmark regression results.

	(1)	(2)	(3)	(4)	(5)	(6)
	DEPTH			SCOPE		
PFTZ	0.0211***	0.0088***	0.0052**	0.1343***	0.0357***	0.0255***
	(6.9172)	(3.5001)	(2.0998)	(11.7224)	(3.7500)	(2.6315)
Size		0.0195***	0.0192***		0.1850***	0.1831***
		(10.0776)	(9.8655)		(26.8959)	(26.6019)
Age		0.0781***	0.0728***		0.1284***	0.1216***
		(7.1154)	(6.6783)		(3.0205)	(2.8727)
Roe		-0.0137**	-0.0130**		-0.0965***	-0.0935***
		(-2.1010)	(-1.9898)		(-3.9638)	(-3.8556)
Cashflow		0.0408***	0.0406***		0.0498	0.0446
		(3.3875)	(3.3757)		(1.2337)	(1.1060)
Growth		0.0007	0.0008		-0.0119*	-0.0110*
		(0.3464)	(0.4195)		(-1.9108)	(-1.7654)
Top1		-0.0427***	-0.0424***		-0.2064***	-0.2070***
		(-3.8238)	(-3.8087)		(-4.5593)	(-4.5943)
PGDP			-0.0163***			-0.0114
			(-2.8878)			(-0.6310)
GI			0.1136***			-0.1802
			(2.9523)			(-1.2555)
FL			0.0014			-0.0504***
			(0.4503)			(-3.7639)
OD			-0.0119***			-0.0802***
			(-4.4665)			(-8.6684)
IS			0.0548**			-0.3232***
			(2.2900)			(-3.3624)
_cons	0.0411***	-0.5960***	-0.4244***	0.4011***	-3.9602***	-3.4749***
	(38.3244)	(-11.6974)	(-5.2339)	(89.5427)	(-21.7424)	(-12.4623)
Year	YES	YES	YES	YES	YES	YES
Firm	YES	YES	YES	YES	YES	YES
Industry	YES	YES	YES	YES	YES	YES
N	24159	24159	24159	24159	24159	24159
R-sq	0.0807	0.6256	0.6268	0.1555	0.7251	0.7264

Notes: The value in parentheses is the t-statistics;

***, **, and * are significant at 1%, 5% and 10% levels, respectively.

### 5.2. Parallel-trend analysis

**Fig 2:**
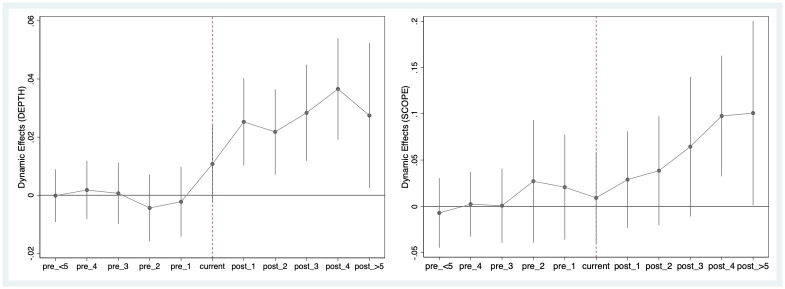
Parallel-trend tests.

The validity of our main results in Table 2 relies on a crucial prerequisite must be met, which is that the trend between the treatment group and the control group is similar before policy implementation. Hence, it becomes imperative to ascertain whether DEPTH and SCOPE of two groups adhere to the parallel trend assumption before establishing PFTZs. Following the research of [22], we adopted the event study method to examine the dynamic effects of establishment of PFTZs on DEPTH and/or SCOPE. Fig 2 plots the dynamic effects of DEPTH and SCOPE, respectively. Specifically, before establishment of PFTZs (the left side of the red dotted line), the coefficients of PFTZ (black dots) are mostly distributed near the 0 value and not significant at the 95% confidence interval. The point estimates mean that there was little to no pre-trend for either DEPTH or SCOPE before establishing PFTZs, thereby passing the parallel-trend test. Furthermore, in Fig 2 (the right of the red dashed line), it is discernible that PFTZs’ implementation triggers a significant influence on DEPTH, which emerges in the immediate year following the policy shock and persists in an increasing trend. This indicates that the positive effect of PFTZs on DEPTH is persistent. Conversely, the policy effect of PFTZs on SCOPE is significantly delayed, becoming evident 4–5 years following establishment of PFTZs. From a temporal trend standpoint, PFTZ establishment is marked by a two-year time lag in its positive effect on SCOPE compared with DEPTH. This implies that enterprises progressively transition from overseas sales to overseas investments, an evolution that happens after they obtain a comprehensive understanding of overseas markets and accumulate sufficient experience abroad. This finding mirrors that of [60], corroborating the idea of internationalization as a gradual process embedded in knowledge learning and experience accumulation.

### 5.3. Robustness tests

#### 5.3.1. Placebo tests

**Fig 3:**
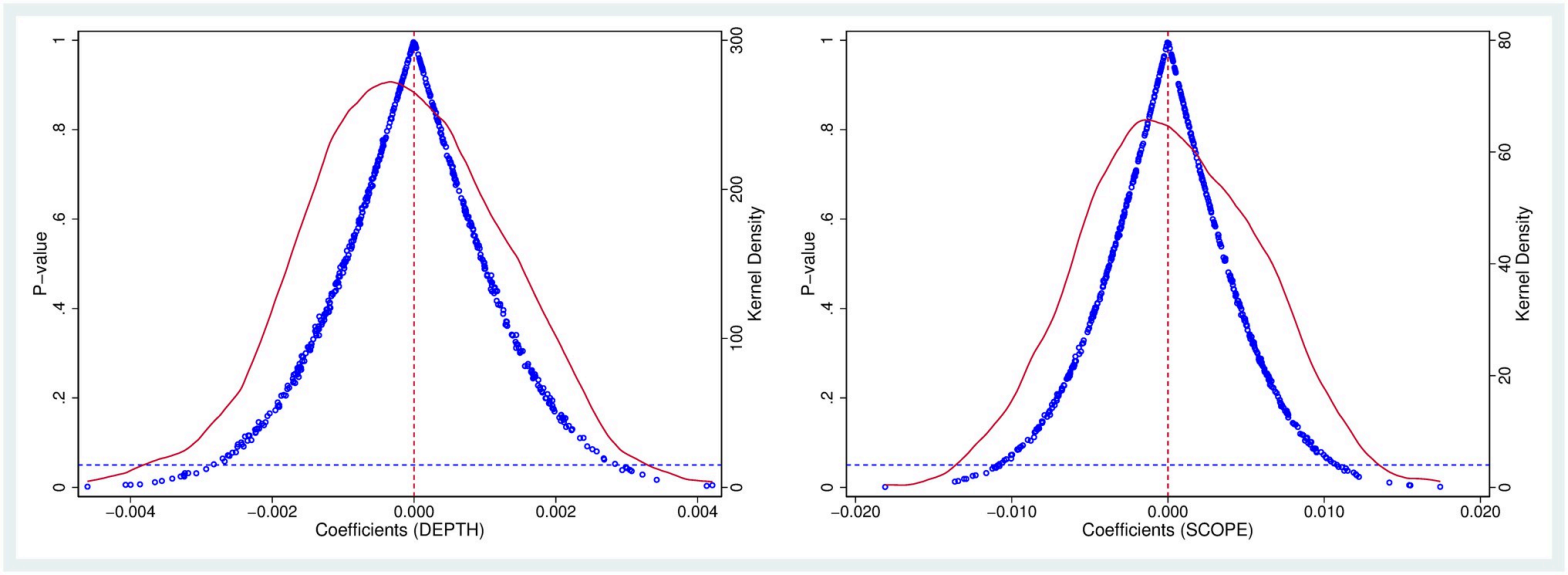
Placebo tests.

To mitigate potential endogeneity and diminish the impact of unobserved omitted variables that might affect benchmark regression results, we reference the approach of [41], which involves conducting a placebo test to affirm reliability. This process begins with construction of a series of false PFTZs by randomly selecting 36 cities as PFTZ pilot cities. Following this, the actual PFTZ is replaced by the false PFTZ in Eq (1) for re-estimation. Lastly, the above steps are replicated 500 times, and estimated coefficients are extracted. In an ideal scenario, the false PFTZ should exert no influence on the international expansion of enterprises, meaning that coefficients of the false PFTZ ought not to significantly deviate from 0. Evidently, the coefficients of the false PFTZ follow a nearly normal distribution, with the majority of samples congregated near 0 (red dotted line) and have statistically insignificant p-values (blue hollow dots). The substantial difference observed between coefficients of the real PFTZ (i.e., 0.0052 & 0.0255) and the false PFTZ (as in Fig 3) indirectly substantiates the validity of the placebo test. These results imply that the influence of other unobservable factors on benchmark regression results can be efficiently excluded.

#### 5.3.2. PSM-DID tests

The preceding results demonstrated that the efficacy of the DID method in differentiating between the policy effect of PFTZs and time trends, it fell short of adequately addressing variable selectivity issues as a consequence of sample selection bias. To curb the endogeneity problem emanating from sample selection bias, we borrow from the study of [8], and employ the PSM-DID method for re-estimation. In particular, all control variables and industry dummy variables are treated as covariates and are matched on a year-by-year basis using the 1:1 nearest neighbour matching method, which mitigates the ‘self-matching’ issue caused by transformation of full-sample panel data from varied periods into cross-sectional data [61]. The balance test of PSM is shown in the S2 Table. Then the matched sample observations that satisfy the common support range are re-estimated based on Eq (1). The results in Columns (1) and (2) of Table 3 show that coefficients of PFTZ are significantly positive, thereby reinforcing the credibility of the benchmark regression results.

**Table 3:** Robustness test.

	(1)	(2)	(3)	(4)	(5)	(6)
	PSM-DID		Exclude BRI interference		Urban characteristics	
	DEPTH	SCOPE	DEPTH	SCOPE	DEPTH	SCOPE
PFTZ	0.0047*	0.0240**	0.0083***	0.0191*	0.0053**	0.0247**
	(1.8468)	(2.4479)	(3.1971)	(1.8960)	(2.1313)	(2.5506)
_cons	-0.4045***	-3.3066***	-0.4325***	-3.4580***	-0.4306***	-3.6382***
	(-4.9926)	(-11.8036)	(-5.3329)	(-12.3864)	(-5.2136)	(-12.2795)
CVs	YES	YES	YES	YES	YES	YES
Year	YES	YES	YES	YES	YES	YES
Firm	YES	YES	YES	YES	YES	YES
Industry	YES	YES	YES	YES	YES	YES
N	14689	14689	24159	24159	24159	24159
R-sq	0.6260	0.7247	0.6278	0.7246	0.6269	0.7265

Notes: The value in parentheses is the t-statistics;

***, **, and * are significant at 1%, 5% and 10% levels, respectively.

#### 5.3.3. Excluding other policy interference

Given that other policies may potentially influence the benchmark regression results during the sample period, it is crucial to identify and eliminate such effects. Upon thorough scrutiny, it was discerned that the BRI, launched by China in 2013, might have impacted international expansion of enterprises within the same period [62]. To eliminate potential interference from the BRI, we add a dummy variable BRI that is equal to 1 if the city *c* where the enterprise is based, happens to be a crucial node city of the BRI and the year is 2013 or after, and 0 otherwise into Eq (1) for re-estimation. The results in Columns (3) and (4) of Table 3 indicate that the coefficients of PFTZ remain significantly positive, suggesting that the estimated results maintain consistency with benchmark regression results, even after controlling for the influence of the BRI.

#### 5.3.4. Accounting for differences in urban characteristics

As a quasi-natural experiment, the ideal conditions for applying the DID method to evaluate policy effects rests on random selection of treatment and control groups. This signifies that the choice of pilot cities and non-pilot cities should be arbitrary, although this condition is often not met in practice. When determining the list of pilot cities, the government typically factors in a host of intertwined elements such as the level of ‘opening up’, political positioning, social development level, geographical location and population density. Over time, these inherent differences in urban characteristics across cities may exert varying influences on the international expansion of local enterprises, leading to potential estimation bias. Hence, we adopt methods introduced in [63, 64], incorporating the cross-product term of urban characteristic disparities and linear trends over time in Eq (2).

$$\begin{array}{c} DEPTH_{it}/SCOPE_{it}=\beta_{0}+\beta_{1}PFTZ_{ct}+\beta_{2}X_{ict}+C_{c}\times Trend_{t}+\gamma_{i}+\mu_{t}+\eta_{j}+\varepsilon_{it}, \end{array}$$ (2)

Where *C*_*c*_ represents a series of different urban characteristics differences for each city. For this study, we employ proxy variables to capture these distinctions, including whether the city is an economically special zone (Spe), a provincial capital city (Cap), a municipality directly under the control of the central government (Mun), a coastal city (Coa), and located east of the Hu Huanyong Line (Hhy). The *Trend*_*t*_ symbolizes the linear trends over time; and other symbols remain consistent with Eq (1). By incorporating the *C*_*c*_×Trend_t_, we effectively control for the impact of the urban characteristics on international expansion of enterprises over time. The results in Columns (3) and (4) of Table 3 indicate that the coefficients of PFTZ remain significantly positive after the set of cross-product term of urban characteristic differences and linear trends over time are added. Hence, the benchmark regression results are robust.

### 5.4. Mechanism tests

The benchmark regression results presented above provide empirical evidence for a policy effect of PFTZs. However, the specific underlying mechanisms for this relationship are still not well understood and remain somewhat of a ‘black box’. In this section, we use the methodology of [22] to further examine the specific impact mechanisms from three aspects: technology effect, competition effect, and resource allocation effect. Results of the mechanism test are presented in Table 4.

**Table 4:** Mechanism test results.

	(1)	(2)	(3)	(4)	(5)	(6)
	DT	TFP	ME	SA	DEPTH	SCOPE
PFTZ×OWE					0.0812***	0.1813**
					(3.4593)	(2.3239)
PFTZ	0.1031***	0.0235***	0.0035***	0.0058***	0.0040	0.0223**
	(5.5178)	(2.7351)	(3.0035)	(3.5291)	(1.6351)	(2.3284)
OWE					0.0554***	0.3034***
					(4.0476)	(6.2036)
_cons	-4.1088***	-5.5065***	-0.5138***	-3.4198***	-0.4212***	-3.4600***
	(-7.3334)	(-20.8405)	(-15.7033)	(-58.0597)	(-5.2067)	(-12.4320)
CVs	YES	YES	YES	YES	YES	YES
Year	YES	YES	YES	YES	YES	YES
Firm	YES	YES	YES	YES	YES	YES
Industry	YES	YES	YES	YES	YES	YES
N	24159	24159	24159	24159	24159	24159
R-sq	0.7931	0.9362	0.7307	0.9560	0.6278	0.6278

Notes: The value in parentheses is the t-statistics;

***, **, and * are significant at 1%, 5% and 10% levels, respectively.

Firstly, we tested the channel mechanism of the technology effect. The result in Column (1) of Table 3 shows that the coefficient of PFTZ is significantly positive, suggesting that PFTZs can promote DT. It has been established that DT can significantly promote international expansion of enterprises [65], therefore DT acts as a transmission channel between PFTZs and international expansion of enterprises. This result verifies the technology effect and confirms H2.

Secondly, we tested the channel mechanism for the competition effect. The results in Column (2) of Table 4 show that the coefficient of PFTZ is significantly positive, suggesting that PFTZs can significantly enhance TFP. Previous studies by [66, 67] have provided evidence that TFP can promote international expansion of enterprises. Therefore, we consider TFP as a transmission channel between PFTZs and international expansion of enterprises. Additionally, we believe that establishment of PFTZs can also promote international expansion of enterprises by improving ME. The result in Column (3) of Table 4 shows that the coefficient of PFTZ is significantly positive, indicating that PFTZs can significantly improve ME. [68] has previously demonstrated that ME can facilitate international expansion of enterprises. Therefore, we confirm that ME can act as a transmission channel between PFTZs and international expansion of enterprises. In summary, these results validate the competition effect and confirm H3.

Thirdly, we test the channel mechanism and the moderating mechanism for any resource allocation effect. The result in Column (4) of Table 4 shows that the coefficient of PFTZ is significantly positive, indicating that the establishment of PFTZs can alleviate FC. Previous research by [69] has demonstrated that enterprises’ financing constraints hinder international expansion. Therefore, we postulate that FC is a transmission channel between PFTZs and international expansion of enterprises. Columns (5) and (6) of Table 4 show that the coefficients of PFTZ×OWE are significantly positive, which means that senior managers with overseas work experience can positively moderate the impact of establishment of PFTZs on international expansion of enterprises. That is, a high proportion of senior managers with overseas work experience can strengthen the promoting effect of PFTZ on international expansion of enterprises. Therefore, the resource allocation effect is verified, and both H4a and H4b are supported.

### 5.5. Expansion analysis

#### 5.5.1. The linkage effect of PFTZs and the BRI

China Pilot Free Trade Zones and the Belt and Road Initiative are contemporary platforms for China’s expanded opening up. Development plans of the State Council for the Guangdong and Fujian PFTZs emphasized their role as key hubs and core areas in the 21st Century Maritime Silk Road. These plans aimed to create a new highland of open cooperation with nations and regions along the route. Similarly, plans for Henan, Chongqing and Shaanxi PFTZs prioritized their evolution as vital transportation hubs to bolster construction of the BRI. It is evident that there is a clear alignment between establishment of PFTZs and the objectives of the BRI. Hence, it is worthwhile to explore whether this alignment fosters a linkage effect on international expansion of enterprises. To delve into this, we devised Eq (3).

$$\begin{array}{c} DEPTH_{it}/SCOPE_{it}=\beta_{0}+\beta_{1}PFTZ_{ct}\times BRT_{ct}+\beta_{2}X_{ict}+\gamma_{i}+\mu_{t}+\eta_{j}+\varepsilon_{it} \end{array}$$ (3)

In Eq (3), the coefficients of the interaction term *PFTZ*_*ct*_×*BRI*_*ct*_ are of particular interest, and other symbols are the same as in Eq (1). The results in Columns (1) and (2) in Table 5 show that coefficients of PFTZ×BRI are significantly positive, and the value clearly increases. This suggests a genuine linkage effect between PFTZs and the BRI. Specifically, when a city is designated both as a PFTZ and a critical node city along the BRI, the promotion effect of establishment of PFTZs on international expansion of enterprises is significantly amplified.

**Table 5:** Interactive effect and radiation effect.

	(1)	(2)	(3)	(4)
	Linkage effect		Radiation effect	
	DEPTH	SCOPE	DEPTH	SCOPE
PFTZ×BRI	0.0069**	0.0328***		
	(2.5031)	(3.1586)		
APFTZ			0.0060**	0.0317***
			(2.5089)	(3.5597)
_cons	-0.4357***	-3.5264***	-0.4269***	-3.4960***
	(-5.3638)	(-12.6591)	(-5.2325)	(-12.5503)
CVs	YES	YES	YES	YES
Year	YES	YES	YES	YES
Firm	YES	YES	YES	YES
Industry	YES	YES	YES	YES
N	24159	24159	24159	24159
R2	0.6268	0.7264	0.6268	0.7264

Notes: The value in parentheses is the t-statistics;

***, **, and * are significant at 1%, 5% and 10% levels, respectively.

#### 5.5.2. The radiation effect of PFTZs

PFTZs as part of the national strategic deployment, not only affects the economic growth of pilot cities but also plays an important role in fostering coordinated regional economic development. Therefore, it is crucial to comprehensively consider the potential spatial spillover effects of PFTZs on surrounding non-pilot cities. As an institutional open ‘experimental field’, PFTZs have taken on the role of innovation pioneers. They leverage their institutional advantages to serve as a ‘demonstration’ for adjacent cities, replicating and promoting the achievements of institutional innovation, driving industrial clusters and generating a positive ‘radiation effect’. However, establishment of PFTZs also provides them with certain unique advantages such as preferential policies or deeper levels of institutional innovation. Consequently, enterprises located outside PFTZs may opt to transfer their registered offices or relocate their factories to PFTZs to maximize benefits, thereby generating a negative ‘siphon effect’ on the economic activities of adjacent non-pilot cities. However, whether establishment of PFTZs impacts international expansion of enterprises in adjacent cities remains to be determined. Based on this, we formulated Eq (4).

$$\begin{array}{c} DEPTH_{it}/SCOPE_{it}=\beta_{0}+\beta_{1}APFTZ_{ct}+\beta_{2}X_{ict}+\gamma_{i}+\mu_{t}+\eta_{j}+\varepsilon_{it} \end{array}$$ (4)

In Eq (4), *APFTZ*_*ct*_ is a dummy variable that equals 1 if the city where the enterprise is based is adjacent to city *c* that set up a PFTZ in year *t*, and 0 otherwise; other symbols are consistent with Eq (1). For example, Guangzhou City is adjacent to Huizhou City. Guangzhou City was set up as a PFTZ in 2015, hence, for Huizhou City, before 2015, APFTZ is 0; but in 2015 and thereafter, APFTZ is 1. The results in Columns (3) and (4) of Table 5 indicate that the coefficients of APFTZ are significantly positive, suggesting that the establishment of PFTZs contributes to promoting international expansion of enterprises in adjacent cities; that is, the establishment of PFTZs has a radiation effect on the surrounding cities.

#### 5.5.3. Heterogeneity analysis of geographical regions

During the early stages of China’s reform and opening up, the eastern region emerged as a pioneer in aligning with the international market, reaping the benefits of coastal geographic advantages. Therefore, we posit that there are significant differences in the internationalization of enterprises between the eastern and central-western regions, particularly given that enterprises in the eastern region can better leverage the policy of PFTZs due to their historical experience. To verify this, we categorize sample enterprises into two groups based on their registered locations in either the eastern region or the central-western region. Results in Table 6 reveal that the impact of establishment of PFTZs on international expansion of enterprises varied across different geographical regions. Specifically, coefficients of PFTZ are significantly positive in the eastern region, but not in the central-western region. This divergence in results can be attributed to the eastern region’s earlier embrace of market openness and higher level of economic development, which laid a solid foundation for stronger global competitiveness of its enterprises. In stark contrast, the central-western regions, due to their historically limited economic development, a lower degree of integration into the international market, and relatively weak market competitiveness [70], might have been disrupted by the entry of foreign multinational enterprises with strong competitiveness under more liberal conditions. This could potentially lead to local enterprises being compelled to withdraw from the international market. Given these findings, it can be concluded that establishment of PFTZs is more beneficial for the international expansion of enterprises in the eastern region, while it appears to hinder enterprises in the central-western regions.

**Table 6:** Heterogeneity across geographical regions.

	(1)	(2)	(3)	(4)
	Eastern		Central-Western	
	DEPTH	SCOPE	DEPTH	SCOPE
PFTZ	0.0168***	0.0870***	-0.0063	-0.0217
	(4.5523)	(6.7621)	(-1.2031)	(-0.9227)
Constant	-0.4142***	-4.3954***	-0.1547***	-3.5680***
	(-3.7954)	(-12.6631)	(-2.8220)	(-13.5276)
CVs	YES	YES	YES	YES
Year	YES	YES	YES	YES
Firm	YES	YES	YES	YES
Industry	YES	YES	YES	YES
N	16738	16738	7413	7413
R2	0.6325	0.7299	0.6051	0.7035

Notes: The value in parentheses is the t-statistics;

***, **, and * are significant at 1%, 5% and 10% levels, respectively.

## 6. Conclusion and policy implications

As an important institutional innovation, PFTZs have been assigned with the role of becoming a novel window for China’s foreign trade and investment. Our research offers novel evidence on the influence of PFTZs on ‘Going Out’, and contributes to a more comprehensive and deeper understanding of the microeconomic effect of the establishment of PFTZs. The main conclusions are as follows.

Our study finds that PFTZs can significantly promote the international expansion of enterprises, and this conclusion still holds after accounting for and overcoming a number of endogenous influences. Our mechanism analysis results suggest that the establishment of PFTZs can drive the overseas expansion of enterprises by promoting digital transformation, improving total factor productivity and management efficiency, and easing financing constraints. Moreover, senior managers with overseas work experience play a positive role in strengthening this relationship. These findings contribute to expanding [71]’s study on the investment and trade theory of multinational corporations. Further expansion research also found that the impact of the establishment of PFTZs on the overseas expansion of enterprises not only has a strategic linkage effect with the Belt and Road Initiative, but also has a radiating effect on the overseas expansion of enterprises in the surrounding cities, encouraging enterprises in the surrounding cities of the pilot areas to adopt international strategies. These findings provide compelling micro-level evidence for the promotion of China’s ‘Going Out’ strategy through PFTZs, and offer valuable insights for the implementation of gradual internationalization strategies in other emerging economies.

Additionally, we also put forward some suggestions for better leveraging of the role of PFTZs in promoting the ‘Going Out’ strategy.

1. It is essential to further deepen institutional openness to create a platform that promotes robust cross-border trade and investment between enterprises. Governments should take the lead in institutional innovation, deepen the implementation of the establishment of PFTZs, optimise the negative list management system, enhance the interconnection of domestic and foreign capital markets, and improve trade liberalisation and investment facilitation.
2. The government should continue to enhance the attractiveness of PFTZs for technology, capital and talent. This can be achieved by creating industrial and technological clusters based on comparative advantages within the PFTZs, easing financing constraints on enterprises, and attracting high-quality talents with international experience and learning from their technical and management expertise. At the same time, enterprises should make full use of the high-quality resources available within the PFTZs, such as technology, capital and talent, to enhance competitiveness, actively explore overseas markets and pursue new development models.
3. It is essential to optimise regional openness and strengthen alignment with national development strategies and policies. The leading position of the eastern coastal regions should be consolidated, while the opening up of the central-western regions should be accelerated. Each region should enhance exchanges, learning and regional cooperation based on their respective comparative advantages. In addition, interactions between PFTZs and major strategies of other countries, such as the Belt and Road Initiative, the construction of the Guangdong-Hong Kong-Macao Greater Bay Area and the development of the western region, should be guided. An “opening-up” pattern that integrates domestic and international connectivity and promotes mutual support between East and West should be established.

Although this article broadens boundaries for studying the microeconomic effects of PFTZs, it still presents some limitations. Due to the unavailability of more detailed data, we assume all enterprises located in PFTZs have enjoyed policy benefits, whereas in reality, these benefits may vary for specific zones within the pilot cities. In addition, we only conducted a simple comparison of the DPETH and SCOPE, without discussing the potential interweaving relationship between the two dimensions. In future work, we aim to gradually refine and improve our research in this field.

## Supporting information

S1 Table The batches of the establishment of PFTZs. (DOCX)

S2 Table Test of covariate balancing in year-by-year matching method. (DOCX)

S1 Data The digital transformation of enterprises. (DTA)
