# Supplementary material for: Does the establishment of Pilot Free Trade Zones promote international expansion of enterprises? Quasi-natural experimental evidence from China
Source: PLoS One. 2024 Aug 15;19(8):e0308477. doi: 10.1371/journal.pone.0308477 (PMC11326610; doi:10.1371/journal.pone.0308477)
Supplement: S1 Table — (DOCX) [file pone.0308477.s001.docx]

**Appendix S1 Table.**

**S1 Table. The batches of the establishment of PFTZs.**

| Year | Province-level | City-level |
| --- | --- | --- |
| 2013 | Shanghai | Shanghai city |
| 2015 | Tianjin, Guangdong, Fujian | Tianjin city, Shenzhen city, Guangzhou city, Zhuhai city, Xiamen city, Fuzhou city |
| 2017 | Sichuan, Chongqing, Hubei, Shannxi, Liaoning, Zhejiang, Henan | Chengdu city, Luzhou city, Chongqing city, Yichang city, Wuhan city, Xiangyang city, Xian city, Xianyang city, Dalian city, Shenyang city, Yingkou city, Zhoushan city, Zhengzhou city, Luoyang city, Kaifeng city |
| 2018 | Hainan | Haikou city, Sanya city, Sansha city, Danzhou city |
| 2019 | Hebei, Shandong, Yunnan, Jiangsu, Guangxi, Heilongjiang | Shijiazhuang city, Tangshan city, Langfang city, Baoding city, Jinan city, Qingdao city, Yantai city, Kunming city, Suzhou city, Nanjing city, Lianyungang city, Nanning city, Haerbin city |
| 2020 | Beijing, Anhui, Zhejiang, Hunan | Beijing city, Hefei city, Wuhu city, Bengbu city, Hangzhou city, Ningbo city, Jinhua city, Yiwu city, Changsha city, Yueyang city, Chenzhou city |
| 2023 | Xinjiang | Urumchi city, Kashgar city, Khorgos city |
