# Supplementary material for: Does the establishment of Pilot Free Trade Zones promote international expansion of enterprises? Quasi-natural experimental evidence from China
Source: PLoS One. 2024 Aug 15;19(8):e0308477. doi: 10.1371/journal.pone.0308477 (PMC11326610; doi:10.1371/journal.pone.0308477)
Supplement: S2 Table — (DOCX) [file pone.0308477.s002.docx]

**Appendix S2 Table.**

**PSM’s balance test.**

The results presented in S2 Tables (a) and (b) show that, compared to the same year, the absolute values of the covariate coefficients have decreased significantly after matching. Moreover, following the matching process, a majority of the coefficients became statistically insignificant. In addition, the pseudo R-squared values saw a substantial decrease in all regressions. These findings suggest that systematic bias in the covariates between the treatment and control groups was effectively minimized across different years. Consequently, our PSM’s balance test results are considered reliable.

**S2 Table (a). Test of covariate balancing in year-by-year matching method_ Before matching.**

|  | (1) | (2) | (3) | (4) | (5) | (6) | (7) |
| --- | --- | --- | --- | --- | --- | --- | --- |
|  | 2007b | 2008b | 2009b | 2010b | 2011b | 2012b | 2013b |
| Size | 0.0663 | 0.0139 | -0.0234 | -0.0313 | -0.0593 | -0.0424 | -0.0064 |
|  | (1.1854) | (0.2167) | (-0.3927) | (-0.5559) | (-1.1758) | (-0.9372) | (-0.1464) |
| Age | 1.6399*** | 1.2707*** | 1.0275*** | 0.9692*** | 0.7128*** | 0.8579*** | 0.8298*** |
|  | (7.6268) | (4.8749) | (4.5761) | (4.7202) | (4.6215) | (5.4651) | (5.0383) |
| Roe | 0.6710 | 0.5113 | -0.4796 | -0.5555 | -1.4567** | 0.4736 | 0.0134 |
|  | (1.1966) | (0.8703) | (-0.8727) | (-0.6826) | (-2.0491) | (0.6729) | (0.0235) |
| Cashflow | -0.8963 | -1.9849** | -0.5519 | -0.2702 | 1.0518 | -1.4171* | -1.1376 |
|  | (-1.2807) | (-2.0422) | (-0.5820) | (-0.2994) | (1.2961) | (-1.6589) | (-1.3824) |
| Growth | -0.2756*** | -0.0378 | -0.1681 | 0.0696 | -0.0678 | 0.1673 | 0.0576 |
|  | (-2.6441) | (-0.1992) | (-1.0445) | (0.4528) | (-0.3646) | (1.0768) | (0.3521) |
| Top1 | 1.7379*** | 0.8806 | 0.7675 | 0.8003* | 0.7095* | 0.3634 | 0.4626 |
|  | (3.8263) | (1.6400) | (1.5567) | (1.6581) | (1.7157) | (0.9505) | (1.2555) |
| PGDP | -0.2482 | 0.3632 | 0.3690* | 0.4237** | 0.7039*** | 0.8606*** | 1.5287*** |
|  | (-1.4808) | (1.6141) | (1.8349) | (1.9659) | (3.1952) | (4.2361) | (8.3574) |
| GI | -8.7204*** | -4.9055** | -2.6285 | -1.7439 | 1.2364 | 0.0493 | 6.4664*** |
|  | (-4.2777) | (-2.2914) | (-1.3178) | (-0.8603) | (0.7081) | (0.0313) | (3.6257) |
| FL | 1.1149*** | 0.8745*** | 0.7415*** | 0.8237*** | 0.7743*** | 0.5593*** | 0.6537*** |
|  | (7.5496) | (4.7891) | (5.1555) | (5.7486) | (5.5326) | (4.0419) | (5.0470) |
| OD | 0.9380*** | 0.6299*** | 1.0537*** | 0.8259*** | 0.8124*** | 0.9340*** | 0.0006 |
|  | (8.5797) | (4.6309) | (6.7823) | (6.1755) | (6.4954) | (8.0517) | (0.0037) |
| IS | 1.7796** | 1.8655* | 2.3571** | 2.8216*** | 4.0676*** | 3.3624*** | 2.8823*** |
|  | (2.4825) | (1.9519) | (2.4398) | (3.0658) | (4.8753) | (4.0997) | (3.9712) |
| N | 1616 | 954 | 1021 | 1086 | 1358 | 1609 | 1756 |
| Pseudo R^2^ | 0.1620 | 0.1446 | 0.1519 | 0.1385 | 0.1326 | 0.1313 | 0.1500 |

| (8) | (9) | (10) | (11) | (12) | (13) | (14) | (15) |
| --- | --- | --- | --- | --- | --- | --- | --- |
| 2014b | 2015b | 2016b | 2017b | 2018b | 2019b | 2020b | 2021b |
| -0.0184 | 0.0157 | -0.0195 | 0.0062 | 0.0047 | 0.0091 | 0.2138*** | 0.2218*** |
| (-0.4153) | (0.3468) | (-0.4651) | (0.1557) | (0.1347) | (0.2676) | (3.1724) | (3.9922) |
| 0.8465*** | 0.7783*** | 0.9109*** | 0.6251*** | 0.7049*** | 0.7069*** | 1.0343*** | 0.8682*** |
| (4.8749) | (4.2294) | (5.0234) | (3.5536) | (4.3863) | (4.2603) | (3.2294) | (2.9290) |
| 0.1863 | 0.3141 | -0.8727 | -0.8282 | -0.0297 | -0.2063 | -0.3953 | -1.9733*** |
| (0.3647) | (0.6100) | (-1.4296) | (-1.3795) | (-0.1025) | (-0.6697) | (-0.6388) | (-3.7629) |
| -1.5579* | -2.4292*** | -1.8288** | -0.7227 | 0.2662 | -0.1110 | -1.3616 | 0.0520 |
| (-1.8511) | (-2.7157) | (-2.2022) | (-0.9326) | (0.3676) | (-0.1525) | (-1.0470) | (0.0495) |
| -0.2665* | 0.0533 | 0.0115 | -0.0851 | 0.0655 | 0.2217 | 0.0691 | 0.2121 |
| (-1.8027) | (0.5739) | (0.1191) | (-0.7253) | (0.4371) | (1.4176) | (0.3012) | (1.0893) |
| 0.3263 | 0.2287 | 0.2385 | 0.2040 | 0.2174 | 0.1985 | -0.0958 | 1.0385** |
| (0.8759) | (0.5993) | (0.6615) | (0.5988) | (0.6955) | (0.6459) | (-0.1596) | (2.1737) |
| 1.4016*** | 0.9644*** | 0.4742** | 0.4818** | 0.7432*** | 0.3033 | 2.0006*** | -0.2393 |
| (6.2542) | (4.8814) | (1.9847) | (1.9885) | (3.6666) | (1.4878) | (6.3302) | (-0.7950) |
| 0.4998 | -2.8261* | -3.6208** | -3.6553** | -1.8217 | -2.0075 | 3.8507* | -1.0344 |
| (0.3119) | (-1.7788) | (-2.1043) | (-2.1263) | (-1.2646) | (-1.4909) | (1.8245) | (-0.4797) |
| 0.4618*** | 0.5576*** | 0.5762*** | 0.6863*** | 0.5810*** | 0.7943*** | 0.1908 | -0.3318*** |
| (4.0814) | (4.8375) | (5.3577) | (7.7514) | (6.4739) | (7.9538) | (1.5083) | (-3.1937) |
| 0.6662*** | 0.8800*** | 1.8216*** | 1.8816*** | 1.3262*** | 1.5959*** | 5.4433*** | 4.2663*** |
| (4.9398) | (7.2586) | (6.7347) | (6.7817) | (6.6030) | (7.1688) | (10.4728) | (10.5218) |
| 2.0283*** | 2.2515*** | 1.4983* | 2.4159*** | 1.6665** | 0.5950 | -21.4453*** | -15.2656*** |
| (2.6367) | (3.0205) | (1.8879) | (3.0318) | (2.3051) | (0.9835) | (-8.5375) | (-12.1725) |
| 1716 | 1780 | 1961 | 2157 | 2584 | 2589 | 1291 | 1489 |
| 0.1303 | 0.1781 | 0.1443 | 0.1460 | 0.1252 | 0.1214 | 0.4777 | 0.3190 |

**S2 Table (b). Test of covariate balancing in year-by-year matching method_ After matching.**

|  | (1) | (2) | (3) | (4) | (5) | (6) | (7) |
| --- | --- | --- | --- | --- | --- | --- | --- |
|  | 2007a | 2008a | 2009a | 2010a | 2011a | 2012a | 2013a |
| Size | 0.0363 | 0.0890 | 0.0440 | -0.0015 | -0.0350 | -0.0149 | -0.0369 |
|  | (0.5292) | (1.1393) | (0.6284) | (-0.0225) | (-0.5784) | (-0.2714) | (-0.6986) |
| Age | 0.5509** | 0.4011 | 0.3376 | 0.3030 | 0.1168 | 0.0731 | 0.3106* |
|  | (2.3986) | (1.4460) | (1.3780) | (1.3197) | (0.6479) | (0.3979) | (1.6478) |
| Roe | 0.1264 | 0.5705 | 0.0084 | 0.0939 | -0.4898 | 0.2522 | 0.7163 |
|  | (0.2053) | (0.8430) | (0.0138) | (0.1030) | (-0.5950) | (0.3193) | (1.1347) |
| Cashflow | -0.0852 | 0.1545 | 0.9391 | -0.0469 | 0.6170 | -0.2179 | -1.1500 |
|  | (-0.0951) | (0.1415) | (0.8497) | (-0.0462) | (0.6395) | (-0.2148) | (-1.2617) |
| Growth | -0.0558 | -0.1941 | -0.0739 | 0.0995 | 0.0942 | 0.0248 | 0.1189 |
|  | (-0.4534) | (-0.8183) | (-0.3805) | (0.5479) | (0.4303) | (0.1377) | (0.7097) |
| Top1 | 0.0874 | 0.3550 | -0.0833 | -0.0630 | 0.3290 | -0.0381 | -0.0658 |
|  | (0.1716) | (0.5600) | (-0.1493) | (-0.1159) | (0.6735) | (-0.0886) | (-0.1548) |
| PGDP | -0.1144 | 0.2504 | -0.1093 | -0.0818 | 0.4080* | 0.5710** | 0.5234*** |
|  | (-0.5547) | (0.8781) | (-0.4535) | (-0.3082) | (1.6519) | (2.3637) | (2.5996) |
| GI | -1.0364 | 1.6441 | -1.6863 | -0.9167 | 2.5156 | 2.8398 | 1.1470 |
|  | (-0.4241) | (0.5604) | (-0.7103) | (-0.3797) | (1.3037) | (1.5751) | (0.6653) |
| FL | 0.4898*** | 0.0748 | 0.4137*** | 0.3499** | 0.3115** | 0.2666* | 0.1605 |
|  | (2.9305) | (0.3368) | (2.7318) | (2.2287) | (2.1267) | (1.7844) | (1.1547) |
| OD | 0.1039 | 0.0667 | 0.2897 | 0.1381 | -0.0444 | -0.1137 | -0.2695 |
|  | (0.5873) | (0.3399) | (1.1673) | (0.7074) | (-0.2563) | (-0.6015) | (-1.4231) |
| IS | 0.8310 | 0.2896 | 0.5451 | 0.5296 | 0.9717 | 0.4832 | -0.2799 |
|  | (0.9941) | (0.2565) | (0.5405) | (0.5429) | (1.1091) | (0.5573) | (-0.3600) |
| N | 958 | 582 | 655 | 675 | 859 | 1013 | 1085 |
| Pseudo R^2^ | 0.0127 | 0.0149 | 0.0153 | 0.0090 | 0.0103 | 0.0104 | 0.0127 |

| (8) | (9) | (10) | (11) | (12) | (13) | (14) | (15) |
| --- | --- | --- | --- | --- | --- | --- | --- |
| 2014a | 2015a | 2016a | 2017a | 2018a | 2019a | 2020a | 2021a |
| -0.0374 | 0.0200 | -0.0128 | 0.0015 | -0.0461 | 0.0005 | 0.0337 | 0.1032* |
| (-0.7081) | (0.3677) | (-0.2578) | (0.0309) | (-1.1319) | (0.0125) | (0.4010) | (1.6521) |
| 0.2310 | 0.4162* | 0.3907* | 0.2221 | 0.2963 | 0.2626 | 0.4028 | 0.3062 |
| (1.1360) | (1.9535) | (1.8501) | (1.0360) | (1.5743) | (1.3833) | (0.9392) | (0.9186) |
| 0.4112 | -0.1928 | -0.3299 | -0.5255 | 0.0711 | -0.2602 | -0.1887 | -0.6439 |
| (0.6727) | (-0.2980) | (-0.4585) | (-0.7892) | (0.2154) | (-0.7249) | (-0.2188) | (-1.0364) |
| 0.0815 | -1.2370 | -0.4783 | -1.2409 | 1.0873 | -0.3346 | -1.4702 | 0.0305 |
| (0.0819) | (-1.2145) | (-0.5007) | (-1.3014) | (1.2682) | (-0.3992) | (-0.8761) | (0.0235) |
| -0.2098 | 0.0135 | 0.0232 | -0.0847 | -0.0805 | 0.0830 | 0.1745 | 0.0398 |
| (-1.1869) | (0.1288) | (0.2152) | (-0.6546) | (-0.4627) | (0.4816) | (0.6258) | (0.1537) |
| 0.0257 | -0.3225 | -0.0472 | 0.2706 | -0.0078 | 0.0065 | -0.1423 | 0.3031 |
| (0.0576) | (-0.7288) | (-0.1118) | (0.6550) | (-0.0216) | (0.0181) | (-0.2140) | (0.5401) |
| 0.8157*** | 0.4413** | 0.2427 | 0.0995 | 0.3770* | -0.0469 | 0.4492 | -0.5744* |
| (3.2532) | (2.0408) | (1.1159) | (0.4630) | (1.8763) | (-0.2175) | (1.0398) | (-1.7046) |
| 2.8385 | -2.7746* | -0.1765 | -0.6403 | 0.8857 | 0.3938 | -0.9323 | -1.5146 |
| (1.5033) | (-1.6971) | (-0.1123) | (-0.3878) | (0.6337) | (0.2380) | (-0.3866) | (-0.6694) |
| 0.2705* | 0.2483* | 0.2385* | 0.2261* | 0.0577 | 0.2237** | -0.1515 | 0.2780 |
| (1.9083) | (1.8543) | (1.9139) | (1.9342) | (0.5597) | (2.1459) | (-1.0027) | (1.6368) |
| -0.2693 | 0.0621 | 0.2126 | 0.0478 | 0.0011 | 0.4739** | 0.4103 | 0.9455** |
| (-1.4739) | (0.6203) | (0.9260) | (0.2044) | (0.0056) | (2.0656) | (0.5304) | (2.1013) |
| 0.6861 | 0.6979 | -0.2668 | -0.9027 | -1.2172 | -0.4202 | -5.6773** | -0.1312 |
| (0.8009) | (0.8171) | (-0.3126) | (-1.0467) | (-1.5753) | (-0.6042) | (-2.4235) | (-0.0711) |
| 1071 | 1018 | 1181 | 1246 | 1561 | 1657 | 430 | 698 |
| 0.0141 | 0.0153 | 0.0122 | 0.0122 | 0.0126 | 0.0103 | 0.0193 | 0.0147 |
